# From the archives: On DNA maintenance—SWI/SNF chromatin remodeling complexes, DNA damage repair, and transposon excision repair mechanisms

**DOI:** 10.1093/plcell/koae127

**Published:** 2024-04-23

**Authors:** Peng Liu

**Affiliations:** Assistant Features Editor, The Plant Cell, American Society of Plant Biologists; Donald Danforth Plant Science Center, Saint Louis, MO 63146, USA

## 2023: SWI/SNF chromatin remodeling complexes

In eukaryotic cells, genomic DNA wraps around histones, forming densely packed chromatin. For gene expression to occur, chromatin must be opened to allow transcription machinery access. This task is facilitated by various chromatin remodelers, including the ATP-dependent SWItch/Sucrose Non-Fermentable (SWI/SNF) complexes (Han et al. 2015). Although SWI/SNF complexes are evolutionally conserved, Arabidopsis exhibits unique adaptations within these complexes (Fu et al. 2023). Unlike mammals, where different SWI/SNF complexes share the same ATPase, Arabidopsis SWI/SNF complexes use distinct ATPases (BRM, SYD, or MINU1/2), leading to their classification as BAS, SAS, and MAS (BRM-, SYD-, and MINU1/2-associated SWI/SNF complexes) (Guo et al. 2022; Fu et al. 2023). Through immunoprecipitation-mass spectrometry analyses, Fu et al. identified specific components associated with BAS, SAS, and MAS (Fu et al. 2023). They particularly focused on elucidating the function of SWI3D, crucial for SAS assembly. Performing a chromatin immunoprecipitation-sequencing, they analyzed the distribution of SWI/SNF complexes on chromatin. Their findings revealed that SAS and MAS predominantly accumulate near transcription start sites (TSS), whereas BAS is more abundant over gene bodies. Interestingly, there was a notable shift in BAS occupancy toward TSS upon the loss of SAS, although its biological significance remains unexplored. In summary, the work of Fu et al. offers comprehensive insights into the assembly dynamics of 3 SWI/SNF complexes in Arabidopsis, laying a foundation for further investigation into their components and functions.

## 2019: DNA damage repair

Another important group of complexes involved in chromosome organization is the Structural Maintenance of Chromosome (SMC) complexes. Eukaryotes possess 3 heterodimeric complexes: the cohesin complex (SMC1/3), the condensin complex (SMC2/4), and the SMC5/6 complex (Uhlmann 2016). Among them, the SMC5/6 complex coordinates DNA repair by recombination, holding a pivotal role in maintaining genome stability (Watanabe et al. 2009). The SMC5/6 complex is associated with multiple nonstructural element (NSE) subunits, the majority of which had been fully characterized in Arabidopsis. And the last remaining subunit, NSE4, was investigated by Díaz et al. (Díaz et al. 2019). In Arabidopsis, there are 2 *NSE4* homologs (*NSE4A* and *NSE4B*), with genetic analyses indicating that *NSE4A* is more essential than *NSE4B*. Loss-of-function *nse4a* mutants are lethal, whereas *nse4b* mutants exhibit viability comparable to wild-type plants. A partial-loss-of-function *nse4a* mutant is viable but sensitive to DNA damage treatment, consistent with NSE4A's role as a DNA repair factor. Although both NSE4A and NSE4B interact with the same SMC5 subunit, only NSE4A is activated upon induced DNA damage treatment. Further analyses reveal critical involvement of NSE4A during the seed development, suggesting its vital role in maintaining genome integrity during the rapid mitotic divisions of embryogenesis. Collectively, this research revealed the essential function of the NSE4A subunit within the SMC5/6 complex in DNA damage repair, particularly during the plant reproduction stage.

## 1999: Transposon excision repair mechanisms

**Figure 1.**
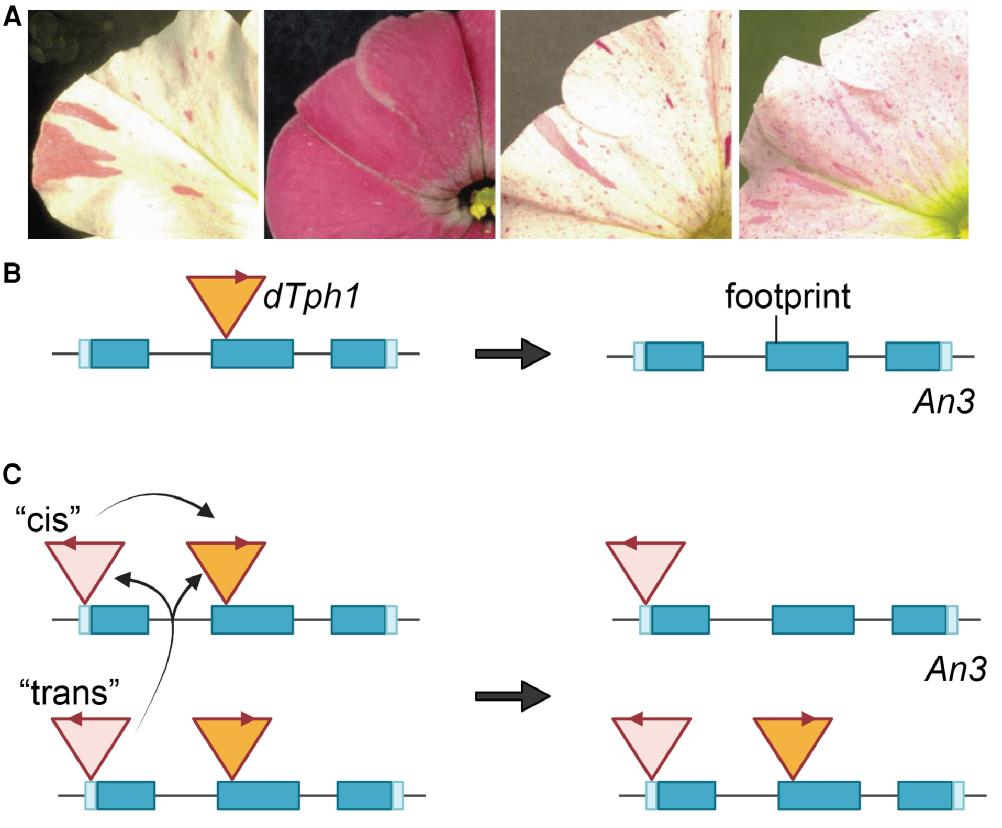
Genetic behaviors of class 1 and class 2 unstable *an3* alleles. **A)** Examples of flower phenotypes from unstable *an3* alleles. **B)** Excision of the *dTph1* transposon from class 1 *an3* allele leaves a footprint. **C)** Excision of the *dTph1* transposon from class 2 *an3* allele excision restores the wild-type sequence. The *dTph1* are shown as triangles with relative orientations. Elimination of the downstream *dTph1* requires interactions from the upstream “cis” and “trans” *dTph1*. Adapted from van Houwelingen et al. (1999), Figures 3A–D and 7A–B.

Lastly, we examine transposable elements, an endogenous inducer of DNA breaks. Transposable elements (or transposons) encompass a diverse set of DNA sequences that can “jump” within a genome through a process called transposition (reviewed by Liu et al. 2022). DNA transposons transpose via a cut-and-paste mechanism, and their excision from the donor site creates DNA breaks that are often not repaired seamlessly. The study of the *dTph1* transposons in petunia provides an interesting example of controlling the excision-repair mechanism during transposition (van Houwelingen et al. 1999). This study used mutant alleles with *dTph1* transposons inserted into the *Anthocyanin3* (*An3*) gene. Functionally inactivating the anthocyanin pigment synthesis pathway, *an3* mutants exhibit a white flower phenotype compared to the usual purple flowers. When an active transposase *Act1* is present, the phenotypes of *an3* mutants become unstable (Fig. 1A) because the transposon can be excised out, thereby restoring the function of the *An3* gene. Interestingly, van Houwelingen et al. found that the same transposon can act differently depending on whether there is an additional copy of transposon. There are 2 distinct classes of *an3* alleles: class 1, where the repair of transposon excision at the donor site is usually nonseamless, leaving a footprint (Fig. 1B); and class 2, where the repair of transposon excision is seamless, perfectly restoring the wild-type sequence (Fig. 1C). The major difference between class 2 and 1 is that class 2 alleles harbor 2 *dTph1* transposons. The excision of the upstream *dTph1* in class 2 behaves like class 1, but the downstream *dTph1* is transposed by a mechanism that seamlessly repairs DNA breaks. Genetics analyses indicate that this novel mechanism requires the presence of both the “cis,” upstream *dTph1*, and its homologous *an3* copy, which is located in “trans” (Fig. 1C). An epigenetic interaction among 3 *dTph1* copies potentially results in the elimination of 1 transposon through a recombination mechanism.

## Dive Curated Terms

The following phenotypic, genotypic, and functional terms are of significance to the work described in this paper:

BRM Gramene: AT2G46020
BRM Araport: AT2G46020

SYD Gramene: AT2G28290
SYD Araport: AT2G28290

MINU1 Gramene: AT3G06010
MINU1 Araport: AT3G06010

MINU2 Gramene: AT5G19310
MINU2 Araport: AT5G19310
